# Pathways towards instability in financial networks

**DOI:** 10.1038/ncomms14416

**Published:** 2017-02-21

**Authors:** Marco Bardoscia, Stefano Battiston, Fabio Caccioli, Guido Caldarelli

**Affiliations:** 1 Department of Banking and Finance, University of Zurich, 8032 Zurich, Switzerland; 2 London Institute for Mathematical Sciences, London W1K 2XF, UK; 3 Department of Computer Science, University College London, London WC1E 6BT, UK; 4 Systemic Risk Centre, London School of Economics and Political Sciences, London WC2A 2AE, UK; 5 IMT School for Advanced Studies, 55100 Lucca, Italy; 6 Institute of Complex Systems CNR, 00185 Rome, Italy

## Abstract

Following the financial crisis of 2007–2008, a deep analogy between the origins of instability in financial systems and complex ecosystems has been pointed out: in both cases, topological features of network structures influence how easily distress can spread within the system. However, in financial network models, the details of how financial institutions interact typically play a decisive role, and a general understanding of precisely how network topology creates instability remains lacking. Here we show how processes that are widely believed to stabilize the financial system, that is, market integration and diversification, can actually drive it towards instability, as they contribute to create cyclical structures which tend to amplify financial distress, thereby undermining systemic stability and making large crises more likely. This result holds irrespective of the details of how institutions interact, showing that policy-relevant analysis of the factors affecting financial stability can be carried out while abstracting away from such details.

## Abstract (web summary)

The spread of instabilities in financial systems, similarly to ecosystems, is influenced by topological features of the underlying network structures. Here the authors show, independently of specific financial models, that market integration and diversification can drive the system towards instability.

Until the 1970s, ecologists widely believed that the stability of an ecosystem was generally enhanced by increasing complexity, as reflected in the presence of a large number of interactions between species. Yet seminal work by May^1^ showed that complexity can actually undermine stability. His analysis of a class of network models indicated that networks with a larger number of interactions (at fixed interaction strengths) were less stable, inspiring ecologists to begin searching for possible new sources of stability in specific topological motifs within food webs. In the wake of the financial crisis of 2007–2008, Haldane and May argued^2^ for the relevance of this insight to the stability of financial systems as well. Indeed, while the pre-crisis literature in economics and finance mostly viewed network complexity as helpful for stability, the application of network theory to finance^3^ has made it clear that complexity can destabilize the financial system^4^,^5^,^6^,^7^.

However, a precise understanding of how network complexity undermines stability has remained elusive. A growing body of work^8^,^9^,^10^,^11^,^12^,^13^,^14^,^15^ carries out stress tests on the financial system by computing the distribution of losses conditional upon a given pattern of shocks. To this end, one must rely on specific assumptions on the nature of the financial contracts and the distress propagation mechanisms. Following^16^,^17^, here we take a different approach: Rather than trying to compute the distribution of losses, we simply identify the conditions under which the system amplifies shocks. This allows to abstract from details on the nature of financial contracts.

In this paper we point out the existence of two general mechanisms that strongly influence the stability of financial networks. In particular, we show that two processes that increase the interaction between banks—market integration, which enlarges the number of banks participating in the financial system, and diversification, which leads to a proliferation of contracts—may lead to instability. Moreover, we show how such instability is associated with the emergence in the network of specific cyclic structures, which amplify financial distress. There are different types of connections between financial institutions, both direct such as interbank loans and indirect such as exposures to common assets^17^,^18^,^19^. Our results are derived in the context of systemic risk emerging from networks of direct exposures between financial institutions (in the following, ‘banks' for brevity), which are modelled as directed weighted networks^20^,^21^,^22^,^23^ and which pose significant scientific challenges and comes with prominent policy and societal implications^24^.

## Results

### Interbank network

While many factors drive systemic risk, the literature has identified two main channels for the propagation of financial distress through direct exposures. The first is known as illiquidity contagion: If banks anticipate that their counterparties may incur losses, they will try to withdraw their liquid funds from them^25^,^26^, inducing them, in turn, to withdraw their funds from their own counterparties. Therefore, distress propagates from lenders to borrowers as their liquidity decreases. The second channel is the deterioration of interbank assets: lenders may reassess the value of their claims towards their borrowers under distress by taking into account the possibility that borrowers might default, and therefore might not be able to meet their obligations. This impacts the balance sheet of the lender, in which assets corresponding to interbank loans will decrease in value. Such accounting practice, called marking-to-market, is enforced by regulatory authorities for certain classes of interbank obligations. In this context, the devaluation of assets effectively generates losses for lenders, which can in turn be transmitted to their creditors^11^,^27^,^28^. Since the process of illiquidity contagion is essentially driven by the anticipation of the potential interbank asset deterioration, here we focus on the latter mechanism only, in line with most of the previous literature^21^,^22^,^27^.

Notice that most works based on the pioneering model of Eisenberg-Noe^27^ conclude that contagion through the network of interbank exposures would be empirically very small^18^,^29^. However, it has been shown that two assumptions in the modelling framework of Eisenberg-Noe imply by construction that interbank contagion has to be very small^30^: the fact that only the event of default affects the value of the obligation and the fact that all remaining assets of defaulting banks are recovered fully and immediately. Indeed, two reasons for why networks of direct exposures can still be important have been discussed in the literature. The first is the fact that counterparty default risk can amplify the so-called ‘balance-sheet contagion'^31^ due to overlapping portfolios^32^. The second reason is that ‘declines in credit quality can propagate losses well before any node has failed'^29^, as indeed modelled in a growing strand of work^11^,^13^,^14^,^33^. This argument finds empirical support in ref. 34, in which it is estimated that two thirds of the losses related to counterparty risk are due to mark-to-market devaluation of assets and one-third to defaults.

The equity *E* of a bank, that is, the difference between its total assets and liabilities, is an important variable in determining the financial health of a bank. In the literature on financial contagion^11^,^27^,^28^, a bank defaults as soon as its equity becomes negative, as it is unlikely that it will be able to repay its debts in full. The ratio between total assets and equity is called leverage and it is a coarse estimate of the riskiness of a bank, as it is related to the maximum loss on the assets that can be absorbed by the equity of the bank. While leverage is usually understood as a single number for each bank, the notion has been recently extended into the concept of leverage matrix^13^, whereby leverage is computed with respect to each specific asset class or counterparty. In particular, for a system of *n* banks here we consider the *n* × *n* interbank leverage matrix Λ, whose elements Λ_*ij*_ are equal to the ratio between the nominal exposure of bank *i* towards bank *j* and the equity of bank *i*. The total interbank leverage of bank *i* is simply equal to 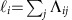. In fact, we will consider an adjusted interbank leverage matrix 
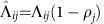
, where *ρ*_*j*_ is the recovery rate of bank *j*, that is, the fraction of its interbank assets recovered by creditors in case of default. Finally, let us denote the relative equity loss of bank *i* at time *t* as *h*_*i*_(*t*)=(*E*_*i*_(0)−*E*_*i*_(*t*))/*E*_*i*_(0).

Starting from basic principles of financial accounting and under mild assumptions on the type of financial contracts among banks, we show that the relative equity loss of bank *i* can be written as a function of the relative equity loss of its counterparties and of the leverage matrix Λ_*ij*_, according to the following dynamics: 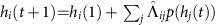, where *p* is the default probability of counterparty *j* as a function of its relative equity loss (see Supplementary Methods for the details). We now briefly argue that it is reasonable to assume that default probabilities are convex functions of the relative equity loss. In fact, the probability of default will be barely affected by small equity losses (as those due to daily fluctuations), while when a bank is close to default, even a small increment in equity losses can make a huge difference. This additional assumption allows us to characterize the stability of the system in terms of 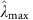 and 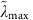, the largest eigenvalues of the matrices 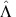 and 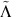, where 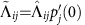. Since 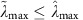, we have three possible regimes: if 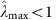 the system is stable, if 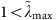 the system is unstable, while if 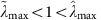 the system could be either stable or unstable (see Supplementary Methods for a full proof). We note that the instability criterion depends on default probabilities, while the stability criterion does not, which is in accordance with the following intuition: it is always possible to make a financial system stable by having probabilities of default that increase slowly enough as equity losses increase.

Despite the considerable body of work on financial contagion, since there is no simple relationship between the topology of a network and *λ*_max_, the study of stability has been seldom carried out in this context. Notable exceptions are ref. 16, in which the stability analysis of the Furfine algorithm^28^ applied to the US CDS market has been conducted, and ref. 17, in which the stability of bipartite networks of overlapping portfolios has been probed through a mapping of the contagion dynamics onto a branching process. By building on these previous analyses, here we quantify the importance of cycles, and we highlight the existence of general mechanisms that might lead to the emergence of instability in the network of mutual exposures between banks.

Our starting point is the definition of pathway towards instability as a sequence of networks (represented here by their weighted adjacency matrices) Λ^(0)^, Λ^(1)^, …, Λ^(*k*)^ such that (i) the dynamics corresponding to Λ^(0)^ is stable for all choices of probabilities of default, (ii) there exist at least one choice of probabilities of default such that the dynamics corresponding to Λ^(*k*)^ is unstable, and (iii) the average interbank leverage is the same for all the networks in the sequence. That the average interbank leverage does not change rules out trivial pathways towards instability; in fact, in the absence of such constraint, it would be easy to build sequences of interbank leverage matrices with larger and larger weights. The aforementioned stability criteria provide a simple way to check if a sequence of networks is a pathway towards instability: it will suffice to check that the largest eigenvalue of 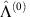 is smaller than one and that the largest eigenvalue of 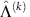 is larger than one. Based on the definition of pathway towards instability, here we show two important effects pertaining to financial instability that had remained uncovered so far and could have profound policy implications. First, even if the individual leverage of banks does not increase, a financial system can turn from stable to unstable as the number of banks increases (that is, the number of nodes in the network grows larger) like during a process of market integration. Second, even if the individual leverage of banks does not increase, a financial system can become unstable as the number of contracts among banks increases (that is, the number of edges in the network increases) like during a process of risk diversification. Notably, in both cases instability appears despite the fact that the assessment that each bank makes of its own risk profile does not change, because individual leverage levels remain constant. This means that market integration and risk diversification can make the system *as a whole* unstable. These results do not imply that such processes are detrimental *per se*, but that financial policies focusing only on individual banks, also known as micro-prudential policies, can have the opposite effect of increasing financial instability if they do not consider the system as a whole. As it will be clear further below, the origin of instability lies in the fact that in both processes banks get increasingly involved in multiple cycles (that is, closed chains) of contracts. Our results suggest to include the eigenvalue analysis of the leverage matrix among the tools to monitor financial stability.

### Emergence of instability

In order to keep the notation agile, in the remainder of the paper we set recovery rates equal to zero, so that 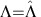. If recovery rates are strictly larger than zero one simply has to compute 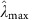 instead of *λ*_max_. The relation between *λ*_max_ and interbank leverage across banks becomes simple if all banks have the same interbank leverage or if the interbank network is a large Erdős-Rényi graph^35^. In the first case, via the Perron-Frobenius theorem, *λ*_max_ is bounded by the smallest and largest sum over the columns of the interbank leverage matrix, that is, precisely by the smallest and largest interbank leverages. Hence, if all banks have the same interbank leverage 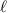, it must be also equal to *λ*_max_. The second case is similar to the May-Wigner theorem about the instability of model ecosystems^1^ in which species interact through a large Erdős-Rényi graph. The main difference is that in our case interactions between banks are described by the leverage matrix Λ, which is non-negative, while the interactions between species in ecosystems are described by a matrix whose elements can have unspecified sign. In the Supplementary Methods we prove that, for *n*→∞, in this case 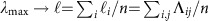, the average interbank leverage across banks. Therefore, in both cases the system is unstable whenever 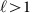.

When relaxing either of the two assumptions (homogeneity of leverage, or large size together with randomness of the graph), finer details of the network structure become important. For instance, because the theorem only holds in the limit of large size graphs, there exist small Erdős-Renyi graphs that are stable although they have 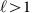. An example of a small size network that is extremely important for policy is the network of the Global Systemically Important Banks^36^, comprising about 30 banks. Let us start from a small and stable Erdős-Renyi graph with 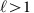 and to connect more banks to the network (by keeping 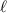 and the number of contracts per bank constant). Eventually, the system will grow large enough to become unstable because the theorem will have to hold in the limit of large graphs (see Supplementary Fig. 1 for an example). This is an example of a previously unreported phenomenon that we call pathways to instability, that is, the existence of trajectories in the space of graphs along which financial networks turn from stable to unstable, although at each point along the trajectory the system satisfies a global constraint on the average interbank leverage. While the theorem above guarantees the existence of pathways towards instability only for Erdős-Rényi graphs, one can perform numerical experiments to investigate additional topologies as well^37^,^38^,^39^. In analogy with Erdős-Rényi graphs, one starts with stable graphs with 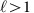, increases the number of banks by keeping both the topology and the average interbank leverage constant, and checks if at the end of the process the graphs become unstable. In Supplementary Figs 2–4 we show that pathways towards instability exist also for regular random graphs, scale-free graphs and core-periphery graphs. The last example is especially relevant, as empirical studies^40^,^41^ have found real interbank networks to be compatible with the core-periphery topology. Therefore, we build realistic models of interbank networks by generating random core-periphery graphs using the parameters in ref. 41. However, interbank exposures are confidential and usually available only to regulators. The information that is publicly available is, for each bank, the total amount of interbank assets and the total amount of interbank liabilities. In order to cope with this problem, several techniques that allow to *reconstruct* exposures based on the limited publicly available information have been developed^42^,^43^,^44^. In particular, we reconstruct interbank exposures using the RAS algorithm^45^, which assigns exposures so that, for each bank, the total interbank assets and the total interbank liabilities match the values reported in their balance sheets (see Supplementary Methods for additional details).

In general, the system is unstable if and only if there exists an unstable strongly connected component (that is, a directed subgraph in which each node is reachable indirectly by any other). The Perron-Frobenius theorem only guarantees that the largest eigenvalue of a strongly connected component is between the minimum and the maximum interbank leverage across banks. Hence, a sufficient condition for instability (stability) is that the interbank leverage of all banks is larger (smaller) than one. However, for the years from 2008 to 2013, the smallest interbank leverage of European banks is very close to zero, while the 95th percentile of its distribution is between 2.5 and 6, meaning that the Perron-Frobenius bounds are not informative enough on the largest eigenvalue, and we need to look more closely at the topology of the network. For instance, for graphs without cycles (that is, directed acyclic graphs, DAGs) *λ*_max_ is always equal to zero, implying that the presence of cycles is a necessary condition for instability (although not sufficient). Intuitively, a cycle amplifies distress propagation if the product of the weights of its edges is larger than one (we refer to this as an individually unstable cycle). Interestingly, a policy recommendation included in Basel III Accords^46^ encourages banks to have the largest single exposure smaller than a fraction of their equity, so that Λ_*ij*_<1 for all *i*, *j*. The policy is thus effective in avoiding this source of instability.

**Figure 1:**
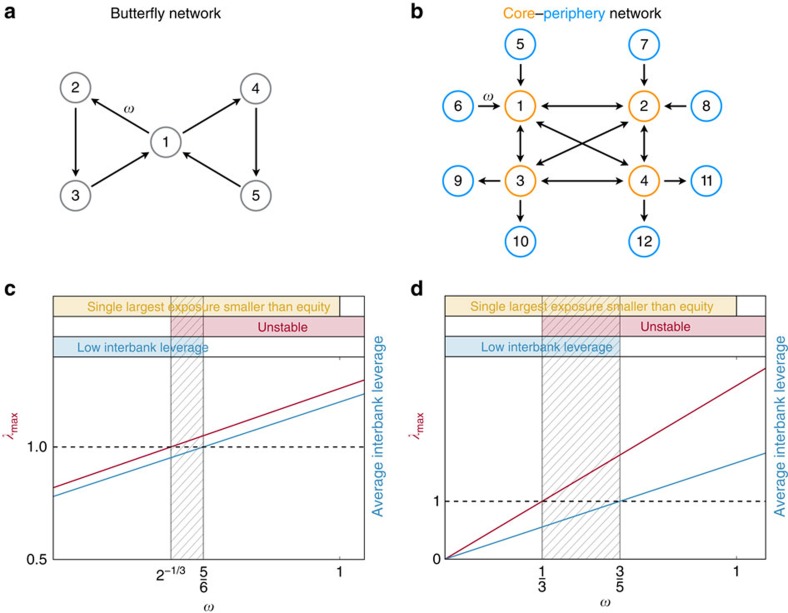
Illustrative stability analysis of two paradigmatic interbank network architectures. The example in **a** is a `butterfly' graph, while the example in **b** has a core-periphery topology: nodes 1, 2, 3 and 4 form a complete core, with the remaining nodes having either only incoming or outgoing edges to the core. For simplicity we set all non-zero elements of the interbank leverage matrix equal to *ω*, implying that the largest single exposure policy is implemented whenever *ω*<1. In **c** and **d** we plot the average interbank leverage (blue line) and *λ*_max_, the largest eigenvalue of the interbank leverage matrix (red line) corresponding to **a** and **b** respectively, as functions of the parameter *w*. The blue region corresponds to an average interbank leverage smaller than one, the yellow region to the largest single exposure smaller than the corresponding equity, while the unstable region is highlighted in red. In both cases there exists a region (shadowed in the figure) in which the following three properties hold: the average interbank leverage is larger than one, the largest single exposure is smaller than the corresponding equity, and yet the network is unstable. Slight modifications of the above examples can also account for tighter constraints on the largest single exposure. For example, even requiring that the largest single exposure is smaller than 15% of the equity (as requested in ref. 46) is not enough to avoid instability in a core-periphery topology with eight nodes in the core.

However, the presence of individually unstable cycles, although sufficient, is not necessary for instability. Consider the two examples in Fig. 1. In particular, the second is a simple case of core-periphery network architecture, a frequently observed pattern in empirical interbank data^40^. In both cases, not only the largest single exposure policy is implemented, but (depending on the value of the parameter *w*) the average interbank leverage can be smaller than one. These two conditions could intuitively suggest that the system is stable. Yet, *λ*_max_ is larger than one and the system is unstable. The reason is that there are banks involved in multiple cycles. More precisely, a sufficient condition for having *λ*_max_>1 is that there exist two integers *i*, *k* such that (Λ^*k*^)_*ii*_>1, that is, that there exists a bank *i* such that the sum, over all the cycles of length *k* from *i* to itself, of the products of the elements of the interbank leverage matrix along each of such cycles is larger than one (we refer to this as a combined unstable cycle). For instance, in the first example of Fig. 1, (Λ^3^)_11_ is larger than one for *ω*>2^−1/3^, and thus there is a range of values where the system is unstable even if the largest single exposure policy is implemented and the average interbank leverage is smaller than 1.

The sufficient condition for instability stated above has important consequences for regulations intended to promote financial stability. Take the case of a bank having a given interbank leverage and at least one exposure larger than its equity. If now the bank is required to implement the largest single exposure policy and it wants to keep its interbank leverage unchanged, it might have to increase the number of its counterparties. On the one hand, this is beneficial because it reduces the exposures towards individual counterparties. On the other hand, it might be detrimental as it could contribute to the creation of new cycles that, even though might be individually stable, are part of a combined unstable cycle. Therefore, a recommendation that targets stability in terms of individual banks can actually lead to instability because it neglects the systemic effect of cycles.

**Figure 2:**
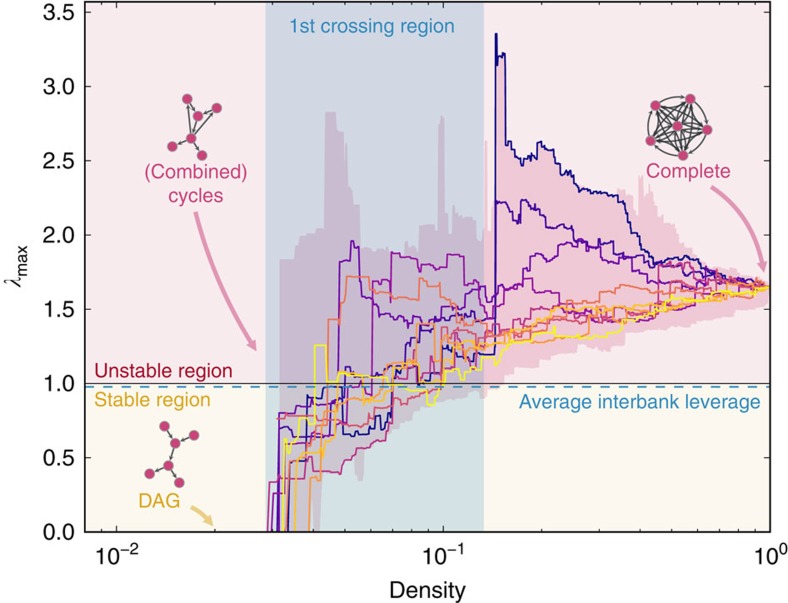
Stability of the network of the top 50 European banks using data from their 2013 balance sheets. We start from a random DAG, that is, a network with no cycles, which is therefore stable. Interbank exposures are assigned with the RAS algorithm so that, for each bank, the total interbank assets and the total interbank liabilities match the value in the balance sheets. We then progressively create new interbank exposures (that is, we randomly add new edges to the interbank network), until all possible exposures have been created (that is, until the interbank network is a complete graph). Every time a new edge is added, we re-balance the interbank exposures so that, for each bank, the total interbank assets and the total interbank liabilities do not change. As a consequence, the degree of diversification in the banking system gradually increases and all interbank leverages do not change. The stability of the network is constantly monitored by re-computing the largest eigenvalue of the interbank leverage matrix every time a new edge is added. We repeat the whole procedure 100 times. We show the contour of all trajectories and highlight a few of them. The first crossing region (in semi-transparent blue) spans the interval of densities of edges across which the networks become unstable for the first time, meaning that combined unstable cycles appear. We can see that densities as low as 3% are sufficient to reach instability. We also plot the average interbank leverage (dashed blue line) for reference. Balance sheet data from the Bankscope database have been initially used in ref. 13.

More in general, increasing the number of contracts in the system is the source of a second type of pathway towards instability. As an empirical illustration of this phenomenon, we consider the balance sheets of the top 50 listed banks in the European Union obtained from the Bankscope data set. We simulate a process in which banks gradually increase the degree of risk diversification by gradually creating exposures towards additional counterparties. We start from an interbank network whose topology is a DAG, which is stable. Exposures are assigned using the RAS algorithm^45^, which ensures that exposures are consistent with balance sheets, that is, that the total interbank assets and the total interbank liabilities of each bank are equal to the values reported in their balance sheets. We then create a new interbank exposure by randomly adding one edge to the graph. After the new edge has been added, interbank exposures are redistributed using the RAS algorithm so that the network is always consistent with the original balance sheets and interbank leverages of all banks do not change. Hence, even though the total amount of interbank exposures of each bank remains constant, as the networks grows denser such exposures are spread across more and more counterparties. As a consequence, the degree of diversification progressively increases. By iterating the steps above we build trajectories in the space of interbank networks whose initial configuration is a random DAG (hence stable) and whose final configuration is a complete graph. We find that, not only the banking system is unstable in this final configuration (that is, once its graph is complete), but actually that the instability kicks in much earlier, when the fraction of existing contracts over all the possible ones is as low as 3% (see Fig. 2 for 2013 balance sheets, and Supplementary Fig. 5 for other years). Moreover, from Fig. 2 we see that trajectories of *λ*_max_ cannot be monotonic and that the critical line can be crossed multiple times, meaning that the system sways between stability and instability, before finally settling into an unstable state. We note that, while the definition of pathways towards instability requires the average interbank leverage to be constant, along the trajectories displayed in Fig. 2 *all* interbank leverages are constant. Therefore, the transitions from stability to instability should be interpreted in an even stronger sense.

**Figure 3:**
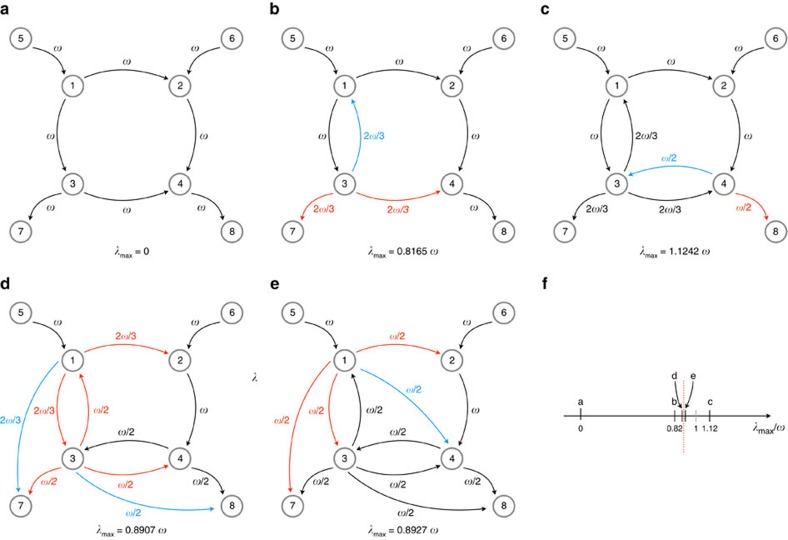
Toy model of an interbank network that oscillates between stability and instability. Going from **a**–**e** we add one or more edges every time, always redistributing the weights so that interbank leverages do not change. Added edges are green, while modified edges are red. The initial network in **a** is a DAG, hence *λ*_max_=0, and for simplicity all edges have the same weight *ω*. Suppose that, as we show in **f**, *ω* is chosen such that *λ*_max_<1 in **d**, but *λ*_max_>1 in **e**. We then have that network in **b** is stable, even though a cycle has appeared. The further addition of one more cycle makes network in **c** unstable. Network in **d** becomes stable again after the addition of two edges, and finally network in **e** is again unstable.

In Fig. 3 we provide a stylized example that helps to connect such changes in the stability of the system to changes in the topology of the network. We start from a DAG, initially setting all non-zero elements of the interbank leverage matrix equal to *w*. We then add one edge at a time, always distributing the interbank leverage of each bank uniformly among the neighbouring (borrowing) banks. *λ*_max_ increases every time a new cycle appears in the system. In contrast, *λ*_max_ decreases whenever a new edge does not lead to the appearance of a new cycle. Intuitively, this behaviour can be explained in the following way. On the one hand, whenever a new cycle appears the possibility for the system to amplify shocks increases. On the other hand, whenever the addition of a new edge does not lead to the creation of a new cycle, the weights of those edges that are part of existing cycles become smaller because interbank leverages are constantly re-balanced, decreasing the ability of those cycles to amplify shocks.

## Discussion

By providing a simple and rigorous mathematical explanation of how network effects arise, our results shed new light on the tension between the two main approaches to financial stability: the so-called microprudential one, focused on ensuring the stability of individual banks, and the macroprudential ones, targeted to the stability of the whole financial system.

We provide examples of sufficient conditions for the onset of instability: when banks establish contracts among each other without taking into account what their counterparties do, they will eventually become even unintentionally part of multiple cycles of contracts, which altogether amplify the effects of shocks. The recovery rate plays an important role, as it impacts directly the critical value of the largest eigenvalue. In turn, the recovery rate can be at least in part controlled with certain financial and monetary policies since it depends on both the quality of the collateral (in the case of secured lending) and on the liquidity of the asset markets. Overall, our findings suggest that financial stability policies need to carefully consider network effects. This can be achieved by computing the largest eigenvalue of the interbank leverage matrix and by comparing it with estimates of the recovery rate.

More specifically, we show the existence of two processes that define trajectories in the space of network configurations which drive financial networks from a stable to an unstable regime. The former consists of implementing processes of market integration (that is, increasing the number of financial institutions) in a growing interbank network with interbank leverage larger than one. The latter consists of increasing the number of contracts among financial institutions. In both cases the risk profile of individual banks (measured by the interbank leverage) does not change, and therefore the emergence of instability is purely related to the structure of the network. This suggests that policies targeted at ensuring financial stability by lowering the risk of individual banks without taking into account the network effects can in fact lead to a higher systemic risk.

Currently the stability of the financial system is assessed by regulatory authorities through stress tests, which are long procedures that last months and are typically run once per year. Stress tests are based on detailed econometric models that require a large number of inputs and the continued cooperation of banks. Even though increasingly sophisticated, usually stress tests consider financial institutions as isolated and neglect the consequences of distress propagation across the network of contracts established among them. Our approach is much more agile, as it allows to gauge the stability of the financial system only through the knowledge of the matrix 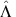. The information required to construct such matrix is: mutual exposures between banks (which regulatory authorities often have access to), equities (which are public) and recovery rates. Recovery rates are not directly measurable, but can be estimated^47^. Moreover, since the largest eigenvalue of the matrix 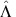 is quickly computed, regulatory authorities can easily analyse a plurality of scenarios corresponding to different potential recovery rates. Finally, while our framework is currently focused on distress propagation due to mark-to-market revaluation of contracts, it is suitable for extensions to additional channels of contagion, such as liquidity shortage due to funds withdrawal. In this case, on the layer corresponding to deterioration of interbank assets the contagion would proceed from borrowers to lenders; on the layer corresponding to liquidity shortages it would proceed from lenders to borrowers. Typically, since relationships between banks might differ from channel to channel, one would construct a multilayered network^48^ with as many layers as the number of channels of contagion. All the layers would be coupled by a single dynamics whose stability could be studied. However, multi-layered networks often exhibit less resilience than single-layered networks^32^,^49^; therefore, as more contagion channels are taken into account, we expect the system to transition more easily to the unstable regime.

### Data availability

All the relevant data are available from the authors on request. Raw data for banks' balance sheet data come from the Bureau van Dijk Bankscope's database.

## Additional information

**How to cite this article:** Bardoscia, M. *et al*. Pathways towards instability in financial networks. *Nat. Commun.* **8,** 14416 doi: 10.1038/ncomms14416 (2017).

**Publisher's note**: Springer Nature remains neutral with regard to jurisdictional claims in published maps and institutional affiliations.

## Supplementary Material

Supplementary Methods Supplementary Methods, Supplementary Figures, Supplementary References
