# Supplementary material for: Pathways towards instability in financial networks
Source: Nat Commun. 2017 Feb 21;8:14416. doi: 10.1038/ncomms14416 (PMC5321761; doi:10.1038/ncomms14416)
Supplement: Supplementary Methods — , Supplementary Figures, Supplementary References [file ncomms14416-s1.pdf]

Supplementary Methods

STABILITY ANALYSIS OF DISTRESS PROPAGATION

In this section we derive, under general mild assumptions, the criteria for carrying out the stability analysis of distress propagation in interbank networks, and we show how the stability of a system of n banks is related to its interbank leverage matrix. The first important ingredient is the balance-sheet consistency at all times t. The balance sheet of a bank is composed by assets and liabilities. The former have positive economic value (e.g. loans towards customers or towards other banks, stocks, derivatives, real estate), while the latter have negative economic value (e.g. deposits, debits towards other banks). In both cases, we distinguish between interbank and external assets or liabilities. Interbank assets (liabilities) are credits (debits) of banks towards other banks, while we call external all other assets and liabilities. We denote by $A_{ij}(t)$ the value at time t of a loan from bank i to bank j, and by $L_{ji}(t)$ the corresponding liability. External assets and liabilities of bank i at time t are denoted by $A_i^{\mathrm{E}}(t)$ and $L_i^{\mathrm{E}}(t)$, respectively. Finally, the equity $E_i(t)$ of bank i at time t is defined as the difference between its assets and liabilities:

$$E_i(t) = A_i^{\mathrm{E}}(t) - L_i^{\mathrm{E}}(t) + \sum_{j=1}^{n} A_{ij}(t) - L_{ij}(t)\,. \tag{1}$$

Assets and liabilities in the balance sheet of a bank depend on time along multiple time-scales. For example, money borrowed from another bank through an interbank loan will remain in the balance sheet until the expiration of the loan. Another example is that deposits (which in this context are external liabilities) might significantly decrease over time as consumers are able to save less money or as other banks become more attractive for depositors. Over shorter time-scales the value of assets can change because banks constantly assess their market value. In other words, banks estimate how much an asset would be worth if it were to be sold today and converted into cash, presumably to pay back other liabilities. Such procedure is known as marking-to-market and it is influenced, among the other things, by considerations about the liquidity of the asset and the probability of default of the counterparty. Let us suppose that bank i issued an interbank loan to bank j for a certain amount of money (the face value); as the probability of default of bank j increases bank i will expect to recover less than the face value and the value of the corresponding interbank assets in its balance sheet will change accordingly.

Here we will focus precisely on such short time-scale dynamics and on a specific asset class: interbank assets and liabilities. Hence, the expiration of contracts (as interbank loans) will be far away in the future and the time dependence of assets and liabilities will be due entirely due to marking-to-market and not to structural changes in the balance sheets. From this perspective it is easy to realise that liabilities do not depend on time. Actually, the fact that bank i might expect to recover less than the face value of its interbank loan towards bank j does not change the fact that bank j still has to pay bank the full face value of the loan, which in its balance sheet appears as an interbank liability.

We follow the assumption, common in the literature on financial contagion, that a bank defaults if its equity becomes negative. The rationale is that the market value of the bank's assets, i.e. the amount of cash that it could be made by liquidating the entire pool of its assets, would not be enough to pay back its liabilities. This assumption implies that balance sheet insolvency is a proxy for default and somehow neglects the liquidity aspects. In fact, a bank with positive equity but no liquidity might default on its payments if it is not able to meet its payment deadlines. However, missing a due payment might or might not trigger a default event, depending on the intricacies of bankruptcy laws, which can vary from country to country. Considering a bank in default when its equity is negative also allows us to abstract from such details.

Interbank loans are established at time $t = 0$, at which point in time their market value $A_{ij}(0)$ will coincide with their face value; otherwise the face value would have been different and would have matched the market value. Let us denote with $p_j(t)$ the probability that bank j defaults before the expiration of its loan (i.e. in the far future) estimated at time t; the bank has obviously not defaulted at time t yet, otherwise its probability of default would be one. At a later time t bank i will estimate that at the expiration of the loan it will recover the face value $A_{ij}(0)$ with probability $1 - p_j(t-1)$ (the probability that bank j will not default) and a smaller value R_{ij} with probability $p_j(t-1)$ (the probability that bank j will default). Therefore, interbank assets will be marked-to-market in the following way:

$$A_{ij}(t) = A_{ij}(0)(1 - p_j(t-1)) + R_{ij}\,p_j(t-1)\,. \tag{2}$$

The time delay from the r.h.s. and the l.h.s. of (2) accounts for the time needed for the information about the probability of default of borrowers to be incorporated into the assessment of lenders.

The scenario we have in mind is to initially stress the system via an exogenous shock to external assets, i.e. $A_i^{\mathrm{E}}(0) \to A_i^{\mathrm{E}}(1) < A_i^{\mathrm{E}}(0)$. Balance sheet consistency (1) implies that such shock will result in losses in equity. We

assume that no additional cash flow (neither positive nor negative) enters the system subsequently. It is reasonable that the probability, estimated at time t, that the default of bank j occurs before the expiration of the loan depends on the equity losses experienced by bank j up to time t. More specifically, we expect that, as the equity losses increase, also the probability of default will increase and, via (2), interbank assets will be devaluated. This, in turn, will lead (again via (1)) to a change in equity. In subsequent rounds external assets do not change and propagation of shocks continues only by iterating such dynamic through the interbank channel. As a consequence, two terms contribute to the loss in equity of bank i between time 0 to time t: the loss in external assets between time 0 and time 1 and the loss in interbank assets up to time t:

$$E_i(0) - E_i(t) = A_i^{\mathrm{E}}(0) - A_i^{\mathrm{E}}(1) + \sum_{j=1}^{n} \left[A_{ij}(0) - R_{ij}\right] p_j(t-1). \tag{3}$$

The aforementioned assumption that a bank defaults if its equity becomes negative implies that the probability of default is a function of the equity. Equivalently, the probability of default can be seen as a function of the equity loss measured with respect to a reference point, as the equity at time zero. By defining $h_i(t)$, the relative loss of equity at time t for bank i, as

$$h_i(t) = \frac{E_i(0) - E_i(t)}{E_i(0)}\,, \tag{4}$$

and:

$$\hat{\Lambda}_{ij} = \frac{A_{ij}(0) - R_{ij}}{E_i(0)}\,, \tag{5}$$

we can re-write (3) as:

$$h_i(t) = h_i(1) + \sum_{j=1}^{n} \hat{\Lambda}_{ij} p_j(h_j(t-1))\,, \tag{6}$$

where we the probability of default of bank j has been written as an explicit function of its relative equity loss h_j. We stress that the assumptions made so far (balance sheets consistency, fair re-evaluation of interbank assets, probability of default as a generic function of the equity) can be considered accounting first principles.

Usually R_{ij}, the amount recovered by the lender bank i in case of default of the borrower bank j, is assumed to be a fraction ρ_j of the face value $A_{ij}(0)$, independent of the lender bank i:

$$\rho_j = \frac{A_{ij}(0)}{R_{ij}} \tag{7}$$

and it is known as recovery rate. Eq. (5) becomes:

$$\hat{\Lambda}_{ij} = \Lambda_{ij}(1 - \rho_j)\,, \tag{8}$$

where

$$\Lambda_{ij} = \frac{A_{ij}(0)}{E_i(0)}\,, \tag{9}$$

is the interbank leverage matrix.

Let us now detail the assumptions on the functions $p_j(h)$. First, such functions map the interval $[0, 1]$ into itself, i.e. $p_j : [0, 1] \to [0, 1]$, as both the relative equity loss and the probability of default take values in such interval. Second, $p_j(0) = 0$, which simply means that the probability of default is zero if no losses have been experienced. Third, $p_j(1) = 1$, which means that when all equity has been wiped out, the probability of default is one. Fourth, p_j are increasing and convex functions. The last two requirements rest on economic motivations. In fact, the larger the equity losses experienced, the larger the probabilities of default. Moreover, the probability of default is expected to increase only marginally for small equity losses (such as those experienced from daily fluctuations of the equity), while, when a bank is close to defaulting ($h_j \simeq 1$) even a small variation in equity can have a large influence on the probability of default. Fifth, we will assume that such functions are differentiable in the interval [0, 1].

Since the relative equity loss cannot become larger than one and given that the probabilities of default are increasing functions of the relative equity loss, the map $\mathbf{h}(t+1) = f(\mathbf{h}(t))$ satisfies the hypotheses of Knaster-Tarski fixed point theorem, meaning that at least a fixed point of such map exists.

Let us now investigate the stability criterion. More precisely we are interested in the condition that will allow, starting from the initial condition $\mathbf{h}(1)$, the limit $\lim_{t\to\infty}\mathbf{h}(t)$ to exist and to be finite. The starting point here is the linear version of the dynamics (6), i.e. with $p_j(h) = h$, for all j:

$$h_i^{\mathrm{L}}(t) = h_i(1) + \sum_{j=1}^{n} \hat{\Lambda}_{ij} h_j^{\mathrm{L}}(t-1)\,, \tag{10}$$

where use the superscript L to explicitly distinguish the relative loss from that computed using (6). The fixed point $\bar{\mathbf{h}}^{\mathrm{L}}$ of (10) is:

$$\bar{\mathbf{h}}^{\mathrm{L}} = (1-\hat{\Lambda})^{-1}\mathbf{h}(1)\,. \tag{11}$$

We will discuss later the significance of the linear dynamics, which at this stage is merely instrumental to our proof. We now observe that $h_j(t) \le h_j^{\mathrm{L}}(t)$, for all j and t. In order to prove it we proceed by induction; first, $\mathbf{h}(1) = \mathbf{h}^{\mathrm{L}}(1)$; second, by assuming $h_j(t-1) \le h_j^{\mathrm{L}}(t-1)$, using the convexity of probability of default we have $p_j(h_j(t-1)) \le h_j^{\mathrm{L}}(t-1)$ and, by using (6) and (10), we easily prove the proposition. Now, if the largest eigenvalue of $\hat{\Lambda}$ is smaller than one, the fixed point $\bar{\mathbf{h}}^{\mathrm{L}}$ will be stable, i.e. $\lim_{t\to\infty}\mathbf{h}^{\mathrm{L}}(t) = \bar{\mathbf{h}}^{\mathrm{L}}$, therefore also the limit $\lim_{t\to\infty}\mathbf{h}(t) = \bar{\mathbf{h}}$ will be finite, and moreover $\bar{\mathbf{h}} \le \bar{\mathbf{h}}^{\mathrm{L}}$. Assuming that shocks are small enough, the fixed point will be within the hypercube $[0,1]\times\ldots\times[0,1]$.

In order to investigate the instability criterion let us assume for a moment that (at least) one fixed point $\bar{\mathbf{h}}$ exist within the the hypercube $[0,1]\times\ldots\times[0,1]$, meaning that:

$$\bar{h}_i = h_i(1) + \sum_j \hat{\Lambda}_{ij} p_j(\bar{h}_j)\,. \tag{12}$$

We can study the dynamics of perturbations around such fixed point by subtracting $\bar{\mathbf{h}}$ from both sides of (6):

$$\begin{aligned} h_i(t) - \bar{h}_i &= h_i(1) - \bar{h}_i + \sum_{j=1}^{n} \hat{\Lambda}_{ij} p_j(h_j(t-1)) \\ &= h_i(1) - \bar{h}_i + \sum_{j=1}^{n} \hat{\Lambda}_{ij} p_j(\bar{h}_j + h_j(t-1) - \bar{h}_j) \\ &\simeq h_i(1) - \bar{h}_i + \sum_{j=1}^{n} \hat{\Lambda}_{ij} \left[p_j(\bar{h}_j) + p_j'(\bar{h}_j)\left(h_j(t-1) - \bar{h}_j\right)\right] \\ &= h_i(1) - \bar{h}_i + \sum_{j=1}^{n} \hat{\Lambda}_{ij} p_j(\bar{h}_j) + \sum_{j=1}^{n} \hat{\Lambda}_{ij} p_j'(\bar{h}_j)\left[h_j(t-1) - \bar{h}_j\right] \\ &= \sum_{j=1}^{n} \hat{\Lambda}_{ij} p_j'(\bar{h}_j)\left[h_j(t-1) - \bar{h}_j\right]\,, \end{aligned} \tag{13}$$

where in the fourth line we have used (12). From the last line of (13) it is clear that $\bar{\mathbf{h}}$ is unstable (stable) if the largest eigenvalue of $\hat{\Lambda}_{ij} p_j'(\bar{h}_j)$ is larger (smaller) than one. We know recall that, since p_j are convex functions, p_j' are increasing functions, implying that $p_j'(0) \le p_j'(\bar{h}_j)$. As a consequence, the largest eigenvalue of

$$\tilde{\Lambda}_{ij} = \hat{\Lambda}_{ij} p_j'(0) \tag{14}$$

is smaller than or equal to the largest eigenvalue of $\hat{\Lambda}_{ij} p_j'(\bar{h}_j)$ (see e.g. Corollary 8.1.19 in [1]). Therefore, if the largest eigenvalue of $\tilde{\Lambda}$ is larger than one, all fixed points will be unstable.

Let us now denote for convenience with $\hat{\lambda}_{\max}$ the largest eigenvalue of $\hat{\Lambda}$ and with $\tilde{\lambda}_{\max}$ the largest eigenvalue of $\tilde{\Lambda}$. Moreover, given that $p_j(0) = 0$, $p_j(1) = 1$, and that p_j are convex, we have that $p_j'(0) < 1$ and thus (again using Corollary 8.1.19 in [1]):

$$\tilde{\lambda}_{\max} \le \hat{\lambda}_{\max}\,. \tag{15}$$

We can therefore have three possible situations. First, $\tilde{\lambda}_{\max} \le \hat{\lambda}_{\max} < 1$, meaning that both the linear dynamics and the non-linear dynamics are stable. Second, $1 < \tilde{\lambda}_{\max} \le \hat{\lambda}_{\max}$ and both the linear dynamics and the non-linear

dynamics are unstable. Third, $\tilde{\lambda}_{\max} < 1 < \hat{\lambda}_{\max}$, in which case the linear dynamics will be unstable, while the non-linear dynamics could be either stable or unstable.

An important observation of that the stability criterion depends on the matrix $\hat{\Lambda}$, which does not contain the probabilities of default. Hence, if we have a network whose $\hat{\lambda}_{\max}$ is smaller than one, the dynamics on that network will be stable, no matter which probabilities of defaults we have chosen. On the contrary, the instability criterion depends on the matrix $\tilde{\Lambda}$, which contains the probabilities of default. If we have a network whose $\tilde{\lambda}_{\max}$ is larger than one, we can always find a local deformation of probabilities of default close to the origin such that $p'_j(0) \to p'_j(0)/(\tilde{\lambda}_{\max} + \epsilon)$, making the system with the new probability of default stable. This result formalises the following intuition. The initial state of the system is such that there are no losses ($\mathbf{h}(0) = 0$) and the probabilities of default are zero ($p_j(0) = 0$). If we now are able to decrease by an arbitrary large amount the rate with which such probabilities of default become larger than zero, we will always be able to make the system stable.

From the vantage point of the previous observation it makes sense to give the following definition. Given the dynamical system in (6), with probabilities of default satisfying the aforementioned hypotheses and with recovery rates ρ_j, we define a pathway towards instability as a sequence of networks $\Lambda^{(0)}, \Lambda^{(1)}, \ldots, \Lambda^{(k)}$ such that i) the dynamics corresponding to $\Lambda^{(0)}$ is stable for all choices of probabilities of default, ii) there exist at least one choice of probabilities of default such that the dynamics corresponding to $\Lambda^{(k)}$ is unstable, and iii) there exist $\ell > 0$, such that $\sum_{ij} \Lambda^{(k)}_{ij}/n = \ell$, for all k. The last requirement implies that the average interbank leverage is the same for all the networks in the sequence. In absence of such requirement, one could easily build trivial pathways towards instability, e.g. by arbitrary increasing the weights of the interbank leverage matrix. Suppose now that we have a sequence of networks and want to check if such sequence is a pathway towards instability. First, we can check if $\hat{\lambda}^{(0)}_{\max}$ (the largest eigenvalue of $\hat{\Lambda}^{(0)}$) is smaller than one, implying that the corresponding dynamics is stable for all choices of probabilities of default. Second, we can check if $\hat{\lambda}^{(k)}_{\max}$ (the largest eigenvalue of $\hat{\Lambda}^{(k)}$) is larger than one, meaning that it exists at least a choice for probability of defaults such that the dynamics is unstable. In fact, $\tilde{\Lambda}^{(k)} = \hat{\Lambda}^{(k)}$ if we choose $p_j(h) = h$, for all j. As a consequence in order to check if a sequence of networks is a pathway towards instability we simply have to compute the largest eigenvalue of $\hat{\Lambda}$ across the sequence of networks.

ADDING NODES

Erdős-Renyi

The crucial thereom that we will exploit is due to Silverstein [2] (Theorem 1.2). In a nutshell, let Λ be a $n \times n$ matrix whose entries are random i.i.d. variables with mean $\mu > 0$ and finite fourth moment. For sufficiently large n, the largest eigenvalue $\lambda_{\max}$ of Λ is:

$$\lambda_{\max} = \frac{1}{n} \sum_{i,j} \Lambda_{ij} + \mathcal{O}(n^{-1/2}) \,. \tag{16}$$

We will now specify the results of the theorem in the case in which the matrix Λ is the weighted adjacency matrix of a random graph. We consider Erdős-Renyi graphs in which $\Lambda_{ij} = C_{ij} W_{ij}$, with $C_{ij} \in \{0, 1\}$ and $W_{ij} \in \mathbb{R}^+$. The variables C_{ij} determine if an edge is present or not and have the bimodal distribution $\rho(C_{ij}) = p\delta(C_{ij} - 1) + (1 - p)\delta(C_{ij})$. The variables W_{ij} are the weights associated with the edges and we leave their distribution unspecified (as long as the fourth moment is finite).

We start with the case in which the network is not sparse, i.e. the case in which the average degree $\bar{k} \equiv \sum_{ij} C_{ij}/n$ is $\bar{k} \simeq \mathcal{O}(n)$, or equivalently $p \simeq \mathcal{O}(1)$ (in the sense that it does not scale with n). Let us define the variables X_i, $i = 1, \ldots n$, as the sums only over columns of Λ, i.e. $X_i = \sum_j C_{ij} W_{ij}$. As C_{ij} and W_{ij} are independent, we have:

$$\langle X_i \rangle = n \langle C_{ij} \rangle \langle W_{ij} \rangle = np \langle W_{ij} \rangle \tag{17a}$$

$$\operatorname{var} X_i = n \operatorname{var}(C_{ij} W_{ij}) = n \left[p \langle W_{ij}^2 \rangle - p^2 \langle W_{ij} \rangle^2 \right] \,. \tag{17b}$$

The next step is to compute $\sum_i X_i/n$. As X_i are i.i.d. with finite variance, using (16) we have that $\lambda_{\max}$ will be normally distributed with

$$\langle \lambda_{\max} \rangle = \frac{1}{n} n \langle X_i \rangle = np \langle W_{ij} \rangle \tag{18a}$$

$$\operatorname{var}\lambda_{\max} = \frac{1}{n^2} n \operatorname{var} X_i = \left[p\langle W_{ij}^2\rangle - p^2\langle W_{ij}\rangle^2\right] , \tag{18b}$$

meaning that the relative fluctuation is $\sqrt{\operatorname{var}\lambda_{\max}}/\langle\lambda_{\max}\rangle \simeq 1/n$.

In the case in which the graph is sparse, i.e. $\bar{k} \simeq \mathcal{O}(1)$ and $p \simeq 1/n$ we know that the degree of each node has a Poisson distribution with mean $\bar{k}$. As a consequence, X_i will have a compound Poisson distribution with

$$\langle X_i\rangle = \bar{k}\langle W_{ij}\rangle \tag{19a}$$

$$\operatorname{var} X_i = \bar{k}\langle W_{ij}^2\rangle \, . \tag{19b}$$

If we now compute the first two moments of $\sum_i X_i/n$ we find that:

$$\langle\lambda_{\max}\rangle = \frac{1}{n} n\langle X_i\rangle = \bar{k}\langle W_{ij}\rangle \tag{20a}$$

$$\operatorname{var}\lambda_{\max} = \frac{1}{n^2} n \operatorname{var} X_i = \frac{\bar{k}\langle W_{ij}^2\rangle}{n} , \tag{20b}$$

meaning that the relative fluctuation is $\sqrt{\operatorname{var}\lambda_{\max}}/\langle\lambda_{\max}\rangle \simeq 1/\sqrt{n}$. Moreover, we can see that the fluctuation on $\langle\lambda_{\max}\rangle$ is of the same order of the correction in (16), therefore we are not able to compute the distribution of $\lambda_{\max}$ in this case.

In the previous derivation we assumed that all entries of the interbank leverage matrix are i.i.d., which is not entirely true. In fact, in our networks a bank cannot extend a loan to itself, meaning that there are no loops (cycles of length one), i.e. the diagonal of the weighted adjacency matrix is filled with zeros. To compute the relative correction on $\langle\lambda_{\max}\rangle$ it will suffice to note that if λ is an eigenvalue of a matrix M, $\lambda - a$ is an eigenvalue of the matrix $M - a\mathbb{I}$. As a consequence, in the case of sparse graphs, we have that $\langle\lambda_{\max}\rangle = np\langle W_{ij}\rangle - p\langle W_{ij}\rangle = (n-1)p\langle W_{ij}\rangle$. Since for graphs without loops $\bar{k} = (n-1)p$, we have that $\langle\lambda_{\max}\rangle = \bar{k}\langle W_{ij}\rangle$. In the case of sparse graphs the correction is already accounted for in (20a), provided that the correct value of $\bar{k}$ is used.

In both cases we have that $\lambda_{\max} = \bar{k}\langle W_{ij}\rangle$, as $n \to \infty$, but with different relative fluctuations. It is worth noting that, when Λ is the matrix of interbank leverage, $\bar{k}\langle W_{ij}\rangle$ is precisely the average interbank leverage ℓ. Therefore, for $n \to \infty$, if $\ell > 1$ the system will be unstable, while if $\ell < 1$ it will be stable. However, if n is not large, fluctuations are relevant, and a system can be stable even if $\ell > 1$, and vice versa. We now provide an example of how adding nodes to such a network can make the system unstable. We start by randomly generating an Erdős-Renyi graph with given p and using an exponential distribution of weights with mean $\langle W_{ij}\rangle$, so that $\ell > 1$, stopping as soon as we find a stable graph. We then proceed to add a new node at a time, by preserving the property that all entries of the weighted adjacency matrix are i.i.d. and by keeping the density of edges (i.e. $\bar{k}$) constant. In fact, if we devised a growth process in which $\bar{k}$ increases, the system would trivially become unstable. We use the following algorithm. Let n be the number of nodes before the addition of a new node i. (i) We randomly form edges from node i and each of the other n nodes with probability p; (ii) we draw a weight from the weight distribution for each of the new outgoing edges from i: (iii) we rescale such weights multiplying them by $(n-1)/n$; (iv) we randomly form edges from each of the other n nodes to node i with probability p; (v) we draw a weight from the weight distribution for each of the new incoming edges for i; (vi) we rescale the weights of all edges starting from the new neighbours of i (including the ones towards node i) so that the sum of all weights of the edges coming out from those nodes do not change after the addition of node i. In Supplementary Figure 1 we see a realisation of such process in which both the density of edges and the average interbank leverage are roughly constant, while $\lambda_{\max}$ becomes larger than one, driving the system towards the instability. Let us note that such algorithm is designed to keep all interbank leverages of the pre-existing nodes constant. However, the probability distribution of single entries of the interbank leverage matrix may vary from a step of the algorithm to the next one. We have checked that the simpler variant in which one keeps the probability distribution of single entries constant and the interbank leverage constant only on average yields the same results.

Regular Random Graphs and Scale-Free Graphs

In the previous section we have used the Silverstein's theorem to prove the existence of a pathway towards instability for growing Erdős-Renyi networks with i.i.d. weights. In the cases in which the theorem does not hold we can still perform numerical experiments to check for the existence of a similar mechanism. The basic idea is to start from a stable graph with average interbank leverage larger than one, to increase the number of its nodes in a way that both

the topology of the network and the average interbank leverage do not change, and to see if during the process the graph becomes unstable. In the remainder of this section we discuss the details of the above process for two specific topologies.

We start from the case of regular random graphs, i.e. graphs in which all nodes have the same in-degree k_{in} and out-degree k_{out}, i.e. $k_{\text{in}} = k_{\text{out}} = k$. In order to generate a directed random regular graph we start by generating an undirected random regular graph by using the algorithm introduced by Steger and Wormald [3]. Clearly, by interpreting such graph as an undirected one, all edges would be reciprocated (meaning that for any edge $i \to j$ there exists also the edge $j \to i$). We therefore perform random edge re-wirings until the fraction of reciprocated edges fell under a certain threshold (we use 0.5 in our numerical experiments). The next step is devise a process to add nodes to a regular random graph such that the new graph is still a regular random graph with the same in-degree and out-degree. To describe how the algorithm works let us add a the new node i. We then randomly select k different pre-existing nodes $\mathcal{N}_{\text{out}} = \{j_1, \ldots, j_k\}$ and, for any of such nodes, we select a random successor to build the set $\mathcal{N}_{\text{in}} = \{l_1, \ldots, l_k\}$, making sure that $\mathcal{N}_{\text{out}} \cap \mathcal{N}_{\text{in}} = \emptyset$. We proceed to add the edges $j_1 \to i, \ldots, j_k \to i$. However, the out-degree of nodes has now increased to $k+1$. Therefore, we remove the edges $j_1 \to l_1, \ldots, j_k \to l_k$ and add the edges $i \to l_1, \ldots, i \to l_k$, so that the in-degrees and out-degrees of all nodes do not change. In order to preserve the interbank leverages of the nodes $j_1, \ldots, j_k$ we simply set $\Lambda_{j_1 i} = \Lambda_{j_1 l_1}, \ldots \Lambda_{j_k i} = \Lambda_{j_1 l_k}$. The interbank leverage of nodes $l_1, \ldots, l_k$ has not changed, since none of their out-coming edges where modified. In order to keep the average interbank leverage ℓ constant, we simply randomly partition the interval $[0, \ell]$ in k sub-intervals and assign the length of the subintervals to the weights $\Lambda_{il_1}, \ldots \Lambda_{il_k}$.

In Supplementary Figure 2 we plot a set of trajectories of the largest eigenvalue of the interbank leverage matrix for growing directed random regular graphs that cross the threshold between stability and instability, showing that also in this case pathways towards instability exist.

We proceed to analyse the case of scale-free graphs. In order to generate random directed scale-free graphs we use the algorithm introduced by Bollobás et al. [4]. Such algorithm implements a growth process that asymptotically leads to directed scale-free graphs. As a consequence, in order to add nodes to our graphs we simply need to iterate it. Due to distribution of the degree of nodes, if we drew all weights from the same distribution, interbank leverages would also have a scale-free distribution whose average would be dominated by the few nodes with a very large degree. If both degrees and interbank leverages have a scale-free distribution unstable cycles appear with a high probability and it is not easy to find a graph that has both average interbank leverage larger than one and the largest eigenvalue smaller than one. Therefore, we tune the distribution of the weights of outgoing edges such that the interbank leverages are, on average, the same. For example, if weights are drawn from an exponential distribution, it will suffice that the mean of the distribution from which the weights of the outgoing edges of any node are drawn is inversely proportional to the out-degree of that node. Every time a new node is added the weights of its outgoing edges are assigned in the same way. However, when a new node is added the algorithm in [4] can also introduce new edges between pre-existing nodes. Therefore, for any node the weights of its pre-existing outgoing edges are rescaled by the ratio between the new and the old degree of the node. Such procedure does not guarantee that the average interbank leverage stays perfectly constant and, in fact, in the numerical experiments we observe that it weakly fluctuates. In order to remove such residual fluctuations, it suffices to simply rescale the weights of all edges by the ratio between the new and the old average interbank leverage.

In Supplementary Figure 3 we plot a set of trajectories of the largest eigenvalue of the interbank leverage matrix for growing scale-free networks that cross the threshold between stability and instability, showing that also in this case pathways towards instability exist.

Core-Periphery with Balance Sheets Data

In this section we consider a more realistic model of interbank networks. We start from the observation that empirical studies [5, 6] have found that real interbank networks are compatible with a core-periphery topology. In such graphs nodes belong to two disjoint sets, the core $\mathcal{C}$ and the periphery $\mathcal{P}$. By properly ordering nodes, the adjacency matrix of such graphs is a block matrix:

$$\left[\begin{array}{c|c} CC & CP \\ \hline PC & PP \end{array}\right],$$

where the block CC contains the edges from nodes in the core to nodes in the core, the block CP contains the edges from nodes in the core to nodes in the periphery, and so on. The diagonal blocks correspond to two different Erdős-Renyi sub-graphs. The off-diagonal blocks correspond to two bipartite random sub-graphs in which edges between nodes in the core and in the periphery are independent and occur with the same probability. Hence, for a graph of

given number of nodes, the core-periphery topology is fully determined by the fraction between nodes in core and nodes in the periphery, and by the densities $\rho_{\rm cc}, \rho_{\rm cp}, \rho_{\rm pc}, \rho_{\rm pp}$ of the edges in the four blocks.

We start by generating a random core-periphery network whose number of nodes matches the number of banks in our dataset and by using the parameters estimated in [5] for the Italian interbank network. In order to assign weights we proceed in the following way. Interbank exposures is considered very sensitive information to which only regulating authorities might have access. In contrast, balance sheets of banks are public, but contain only a partial information about interbank exposure. More specifically, the balance sheet of a bank lists the total interbank assets (i.e. the amount of money lent to other banks) and the total interbank liabilities (i.e. the amount of money borrowed from other banks). Apart from a few selected studies on data held by regulating authorities, the literature on interbank networks approaches this problem by making some assumptions that allow to reconstruct the interbank exposures from the limited information contained in the balance sheets. The choice of the right reconstruction technique is dictated by several considerations, the most important of which is the kind of partial information available. In the case in which the topology and the marginal interbank assets and liabilities are known, exposures can be reconstructed by using the RAS algorithm [7]. The algorithm assigns exposures by assuming that, bank by bank, their distribution maximises the entropy, consistently with the constraints on interbank assets and liabilities.

Generating a core-periphery graph is easy, one simply generates four Erdős-Renyi sub-graph, one for each block. It is slightly more complicated to let the graph grow while the topology does not change. In order to keep the fraction of nodes in the core and in the periphery constant, we assign a new node to the core with probability equal to the desired fraction of nodes in the core and to the periphery with the complementary probability. In order to keep the density of the four blocks we proceed in the following way. Let us denote with $N_{\rm c}$ ($N_{\rm p}$) the number of nodes in the core (periphery) before the new node is added and with $N'_{\rm c}$ ($N'_{\rm p}$) the the number of nodes in the core (periphery) after the new node is added. Analogously, $E_{\rm cc}$ and $E'_{\rm cc}$ are the number of edges between nodes in the core before and after the new node has been added. We use similar notations for the number of edges corresponding to the other blocks. Let us now suppose that a new node is added to the core, therefore in order to keep the density of the core-core block constant we have:

$$\rho_{\rm cc} = \frac{E_{\rm cc}}{N_{\rm c}(N_{\rm c}-1)} = \frac{E'_{\rm cc}}{N'_{\rm c}(N'_{\rm c}-1)} = \frac{E'_{\rm cc}}{(N_{\rm c}+1)N_{\rm c}} \,, \tag{21}$$

meaning that the number of edges to add between nodes belonging to the core is:

$$E'_{\rm cc} - E_{\rm cc} = E_{\rm cc}\frac{N_{\rm c}+1}{N_{\rm c}-1} - E_{\rm cc} = 2E_{\rm cc}\frac{1}{N_{\rm c}-1} = 2\rho_{\rm cc}N_{\rm c} \,. \tag{22}$$

Hence after the node has been added, we also add $2\rho_{\rm cc}N_{\rm c}$ edges randomly chosen among the $2N_c$ possible edges between the new node and all other nodes in the core. In order to keep the density of the core-periphery block constant we have instead:

$$\rho_{\rm cp} = \frac{E_{\rm cp}}{N_{\rm c}N_{\rm p}} = \frac{E'_{\rm cp}}{N'_{\rm c}N'_{\rm p}} = \frac{E'_{\rm cp}}{(N_{\rm c}+1)N_{\rm p}} \,, \tag{23}$$

meaning that the number of edges to add from the core to the periphery is:

$$E'_{\rm cp} - E_{\rm cp} = E_{\rm cp}\frac{N_{\rm c}+1}{N_{\rm c}} - E_{\rm cp} = E_{\rm cp}\frac{1}{N_{\rm c}} = \rho_{\rm cp}N_{\rm p} \,. \tag{24}$$

Hence after the node has been added, we also add $\rho_{\rm cp}N_{\rm cp}$ edges randomly chosen between the possible N_p edges from the new node in the core to the nodes in the periphery. Similarly one finds that number of edges to add from nodes in the periphery and the new node is $\rho_{\rm pc}N_{\rm p}$, while no edges needs to be added between nodes in the periphery. Proceeding in the same way one can derive the number of edges to add in all blocks when the new node belongs to the periphery.

In order to check the existence of pathways towards instability here we proceed in a slightly different way. First, we generate a sequence $\mathcal{G}_0, \ldots, \mathcal{G}_n$ of unweighted core-periphery graphs of increasing number of nodes so that the number of nodes of the final graph $\mathcal{G}_n$ matches the number of banks in our dataset. We assign weights to such graph by using the RAS algorithm (see above). Second, we remove the node in $\mathcal{G}_n$, but not in $\mathcal{G}_{n-1}$ and all its incoming and outgoing edges. This is equivalent to transferring all the weights of the other edges on the unweighted graph $\mathcal{G}_{n-1}$. In this way the topology of the new graph is left unchanged. Third, in order to keep also the average interbank leverage constant we rescale the weights of all edges by the ratio between the new and the old average interbank leverage. We iterate the procedure until we reach the initial graph $\mathcal{G}_0$.

In this case we are traversing the pathway in the opposite directions, from instability to stability. Hence we only keep those sequences such that the graph $\mathcal{G}_n$ is unstable. The reason why in this case we follow the pathway in the

opposite direction is that, in order to proceed in the usual direction (i.e. by adding nodes) we would need to sample a subset of banks in the dataset and to randomly add the other banks, one at a time. However, the average interbank leverage would not remain constant along such process. In Supplementary Figure 4 we plot a set of trajectories of the largest eigenvalue of the interbank leverage matrix for growing core-periphery networks that cross the threshold between instability and stability, showing that also in this case pathways towards instability exist.

Supplementary Figures

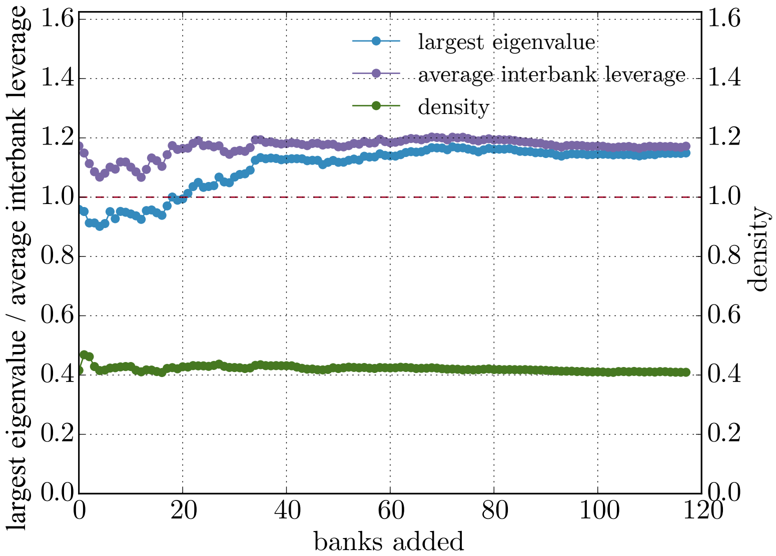

Supplementary Figure 1. **Adding nodes to an Erdős-Renyi graph.** Example of growth process in which a stable network with average interbank leverage larger than one becomes unstable as new banks are added to the system. We stress that the crossing to the unstable regime is genuinely driven by the fact that fluctuations in the asymptotic distribution of $\lambda_{\max}$ shrink as n becomes larger: in fact the density of edges in the network stays roughly constant. Here the initial network has $n = 20$ and the weight distribution is exponential with mean $\simeq 0.79$.

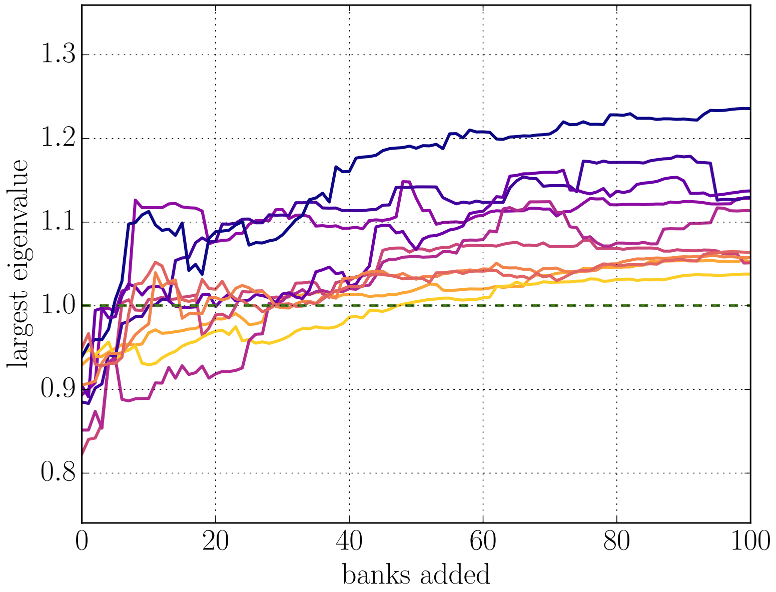

Supplementary Figure 2. **Adding nodes to regular random graphs.** Analogous of Supplementary Figure 1, but for (directed) regular random graphs with in-degree and out-degree equal to ten. Here we show 10 different trajectories of networks crossing from the stable to the unstable regime. For all trajectories both the topology and the average interbank leverage (which is always larger than one) are constant along the whole trajectory. The initial network has $n = 20$ and the weight distribution is exponential with mean $\simeq 0.58$.

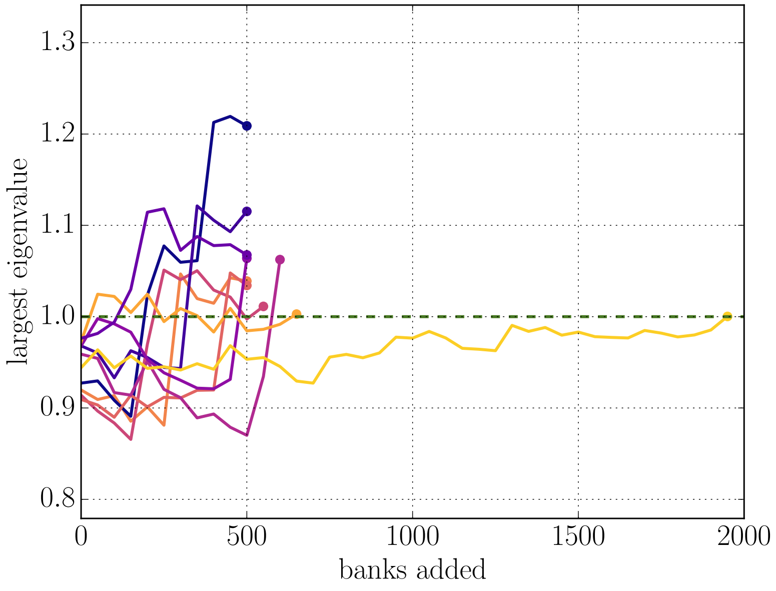

Supplementary Figure 3. **Adding nodes to scale-free graphs.** Analogous of Supplementary Figure 2, but for scale-free graphs with tail exponents for the in-degree and out-degree distributions respectively equal to 2.15 and 2.7. Here we show 10 different trajectories of networks crossing from the stable to the unstable regime. For all trajectories both the topology and the average interbank leverage (which is always larger than one) are constant along the whole trajectory. The initial network has $n = 1000$ and the weight distribution of the outgoing of node i is exponential with mean $2/k_{\text{out}}$. Trajectories are prolonged either until 500 nodes have been added or until the largest eigenvalue becomes larger than one.

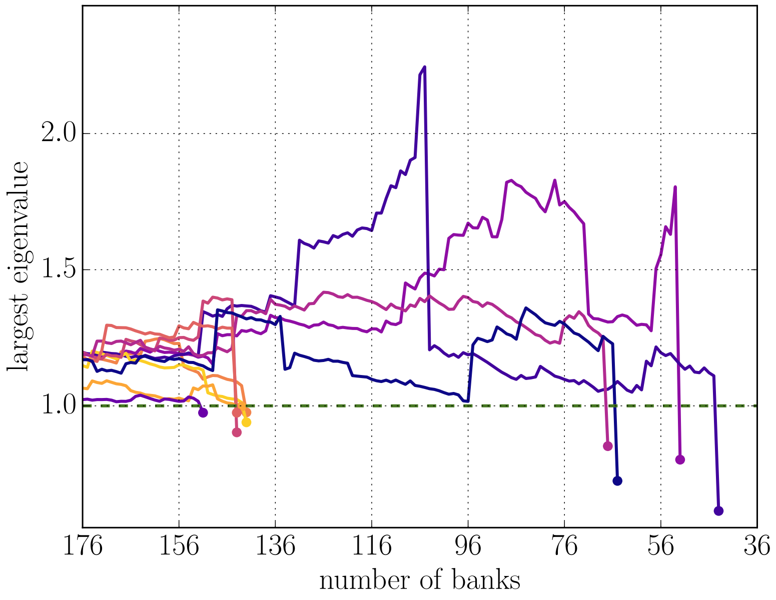

Supplementary Figure 4. **Adding nodes to core-periphery graphs.** Pathway towards instability travelled backwards, i.e. from instability to stability as the number of banks decreases. The topology of graphs is core-periphery with realistic parameters (see [5]). Here we show 10 different trajectories of networks crossing from the unstable to the stable regime. For all trajectories both the topology and the average interbank leverage (which is always larger than one) are constant along the whole trajectory. Initial weights are assigned using the RAS algorithm (see [7]) and are consistent with the balance sheets of the Top 176 European banks for the year 2012 (source: Bankscope dataset). We have chosen the year 2012 as it is the year with the smallest average interbank leverage (hence the year for which it is more difficult to observe unstable networks) larger than one. Trajectories are prolonged until the largest eigenvalue becomes smaller than one. Pathways have been built backwards for technical reasons, namely to keep the average interbank leverage constant while maintaining consistency with real balance sheets (see main text).

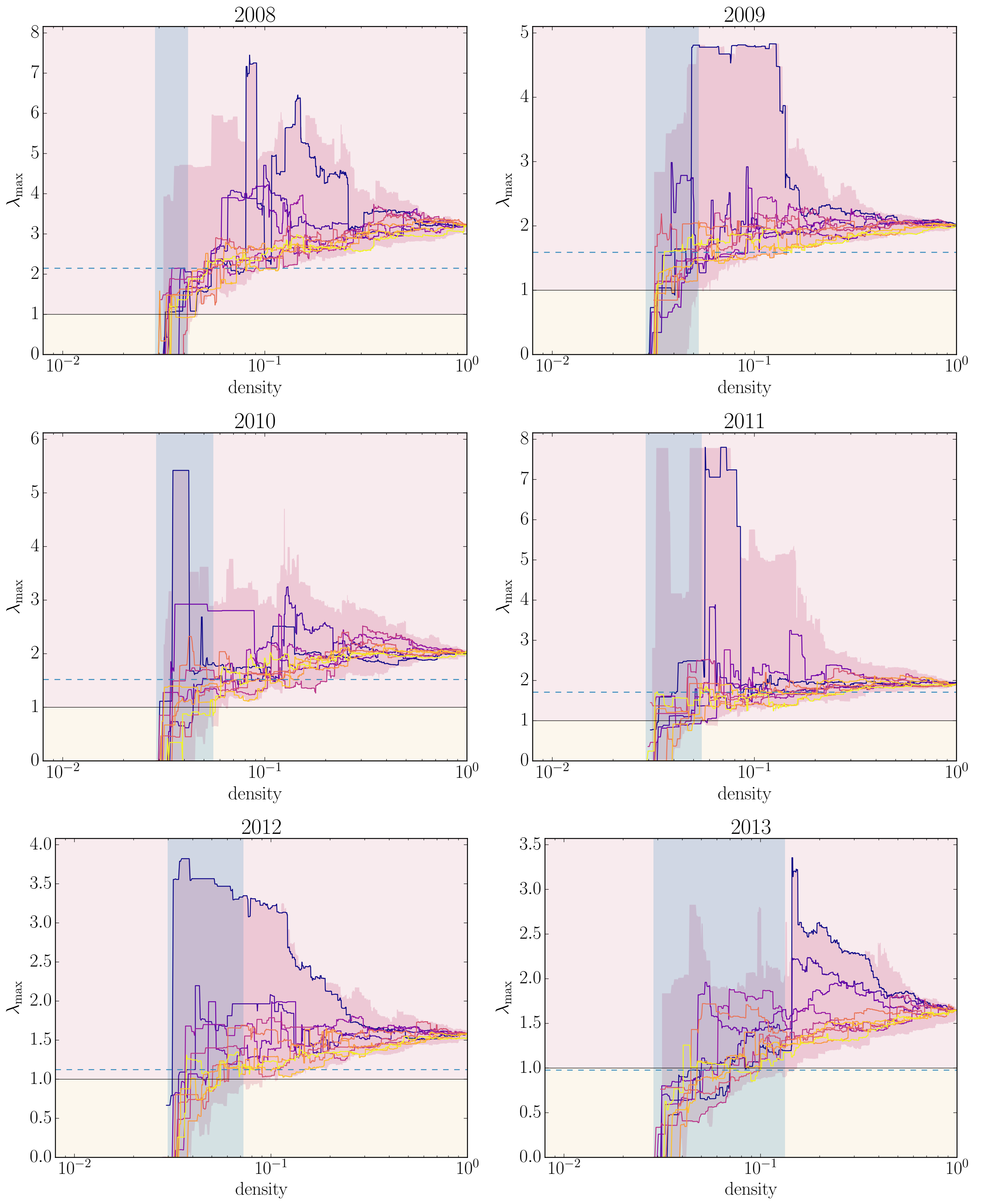

Supplementary Figure 5. **Adding edges to the network of the top 50 European banks.** Analogous of Figure 3 for years from 2008 to 2013. For $\lambda_{max} < 1$ the interbank network is stable (yellow region), while for $\lambda_{max} > 1$ it is unstable (red region). For comparison we also plot (dashed blue line) the average interbank leverage.

Supplementary References

[1] Horn, R. A. & Johnson, C. R. Matrix analysis. (Cambridge University Press, Cambridge, 2012)

[2] Silverstein, J. W. The spectral radii and norms of large dimensional non-central random matrices. *Stochastic Models* **10**(3): 525 – 532 (1994).

[3] Steger, A. & Wormald, N. Generating random regular graphs quickly. *Probability and Computing* **8**: 377 – 396 (1999).

[4] Bollobás, B., Borgs, C., Chayes J., & Riordan, O. Directed scale-free graphs. *Proceedings of the fourteenth annual ACM-SIAM symposium on Discrete algorithms*, 132 – 139 (2003).

[5] Fricke, D. & Lux, T. Core-periphery structure in the overnight money market: evidence from the e-mid trading platform. *Computational Economics* **45**(3), 359 – 395 (2015).

[6] Craig, B. and Von Peter, G. Interbank tiering and money center banks. *Journal of Financial Intermediation* **23**(3), 322 – 347 (2014).

[7] Upper, C. and Worms, A. Estimating bilateral exposures in the German interbank market: Is there a danger of contagion? *European Economic Review* **48**(4), 827 – 849 (2004).
